# The mediation function of resting heart rate in how physical activity improves all-cause mortality: Continuous and automatic measurement *via* cardiac implantable electronic devices

**DOI:** 10.3389/fcvm.2022.928372

**Published:** 2022-09-26

**Authors:** Chendi Cheng, Xue Rong Sun, Keping Chen, Wei Hua, Yangang Su, Wei Xu, Fang Wang, Xiaohan Fan, Yan Dai, Zhimin Liu, Shu Zhang

**Affiliations:** ^1^State Key Laboratory of Cardiovascular Disease, Arrhythmia Center, National Center for Cardiovascular Diseases, Fuwai Hospital, Chinese Academy of Medical Sciences, Peking Union Medical College, Beijing, China; ^2^Department of Cardiology, Shanghai Institute of Cardiovascular Diseases, Zhongshan Hospital, Fudan University, Shanghai, China; ^3^Department of Cardiology, Nanjing Drum Tower Hospital, Nanjing, China; ^4^Department of Cardiology, Shanghai First People’s Hospital, Shanghai Jiao Tong University School of Medicine, Shanghai, China

**Keywords:** physical activity, resting heart rate (RHR), all-cause mortality, remote home monitoring (RHM), mediation effect analysis

## Abstract

**Background:**

Physical activity (PA) and resting heart rate (RHR) are connected with all-cause mortality. Moreover, there was an inverse correlation between PA and RHR. However, the causal relationship between PA, RHR, and long-term mortality has been rarely evaluated and quantified, particularly the mediation effect of RHR in the association between PA and all-cause mortality.

**Objective:**

To describe the relationship between PA and RHR when consistently measured *via* cardiac implantable electronic devices (CIED) and further explore the mediation effect of PA on all-cause mortality through RHR.

**Materials and methods:**

Patients who underwent CIED implantation and received remote home monitoring services were included. During the first 30–60 days after CIED implantation, daily PA and RHR were continuously measured and automatically transmitted by CIED. The primary endpoint was all-cause mortality. The multiple linear regression model was used to confirm the relationship between PA and RHR. The predictive values of both PA and RHR for all-cause mortality were assessed by multivariable Cox proportional hazards models. The causal mediation model was further established to verify and quantify the mediation effect of RHR in the association between PA and all-cause mortality.

**Results:**

A total of 730 patients with CIED were included. The mean daily PA and RHR were 10.7 ± 5.7% and 61.3 ± 9.1 bpm, respectively. During a mean follow-up period of 55.8 months, 187 (26.5%) death was observed. A negative linear relationship between PA and RHR was demonstrated in the multiple regression model (β = −0.260; 95% CI: −0.377 to −0.143, *p* < 0.001). Multivariable Cox proportional hazards analysis showed that both lower levels of PA (HR = 0.907; 95% CI: 0.878–0.936, *p* < 0.001) and higher RHR (HR = 1.016; 95% CI: 1.001–1.032, *P* = 0.031) were independent risk factors of all-cause mortality. Causal mediation analysis further confirmed and quantified the mediation function of RHR in the process of PA improving all-cause mortality (mediation proportion = 3.9%; 95% CI: 0.2–10.0%, *p* = 0.036).

**Conclusion:**

The effects of the higher level of PA on improving life prognosis may be partially mediated through RHR among patients with CIED. It indicates that changes in the autonomic nervous function during postoperative rehabilitation exercises should get more attention.

## Introduction

Physical activity (PA) is defined as any bodily movement produced by the skeletal muscles that need to consume energy (1). Thus, in addition to participating in sports activities with high intensity, several daily activities with low intensity in our life such as part of work, taking a walk, housework, and recreational events can be regarded as part of PA as well. Strong evidence supports that regular PA can potentially prevent major adverse cardiovascular events and all-cause mortality in both healthy people and patients with cardiovascular diseases (CVDs) (2–5). In recent years, the possible existence of the “physical activity paradox” raises further discussion about PA (6–8). However, the high heterogeneity among different studies should also be noted, especially the differences in the measurement of PA. In previous clinical studies, the level of daily PA was assessed by using self-assessment questionnaires, which may be highly subjective and lack uniform evaluation criteria (9, 10). More importantly, the potential underlying mechanism by which PA improves outcomes needs to be further revealed. Previous studies support that regular PA could contribute to enhancing the overall autonomic nervous system (11, 12), which may be one of the reasons for the improved prognosis.

Heart rate, especially resting heart rate (RHR), is an easily accessible indicator in the assessment of sympathetic and parasympathetic activity; thus, it can reflect physical and general fitness (13, 14). Therefore, in several cohorts, RHR may be an alternative when direct measures of PA levels are lacking (15–17). Elevated RHR measured at several single points in time has been shown to be related to more cardiovascular adverse events and a higher risk of all-cause mortality (17–21). Meanwhile, an inverse correlation between PA and RHR was described in previous studies (15–17). Nevertheless, although the relationship between PA, RHR, and all-cause mortality was often mentioned and cited, the causal relationship among them was rarely evaluated and quantified in the same cohort, specifically the mediation effect of RHR in the association between PA and all-cause mortality. Therefore, the aim of this study is to describe the relationship between PA and RHR when consistently measured *via* cardiac implantable electronic devices (CIED) and further explore the mediation function of RHR in how PA improves prognosis.

## Materials and methods

### Study design

The Study of Home Monitoring System Safety and Efficacy in Cardiac Implantable Electronic Device-implanted Patients (SUMMIT) registry is an observational, prospective, and multi-center trial. We retrospectively analyzed archived home monitoring transmission data from the SUMMIT registry. The present study was approved by the ethics committee of Fuwai Hospital (ID: 2010-296) and all other participating organizations. All patients provided written informed consent before entering this study, which complied with the Declaration of Helsinki.

### Patient selection

From May 2010 to April 2014, patients who underwent CIED implantation, including implantable cardioverter defibrillator (ICD) and cardiac resynchronization therapy defibrillator (CRT-D), were included upon meeting two inclusion criteria: (i) CIED with a remote home monitoring system and the system is continuously working during the follow-up, and (ii) data including RHR and PA are available during the target window. The exclusion criteria were as follows: (i) during the target window for RHR measurement, the percentage of daily ventricular pacing in a single-chamber device was more than 10%, or the percentage of average daily atrial and ventricular pacing percentage in a dual-chamber device was more than 10%; (ii) age at CIED implantation was younger than 18 years; (iii) the postoperative survival period was less than 3 months; or (iv) received heart transplantation. Patients were divided into four groups on average based on the baseline level of daily PA. PA group 1 (range, 0.3–6.8%; *n* = 182), PA group 2 (range, 6.9–10.1%; *n* = 182), PA group 3 (range, 10.2–14.5%; *n* = 183), and PA group 4 (range, 14.5–33.3%; *n* = 183).

### Data measurement and collection

Data on the patients’ demographic variables (e.g., gender, age at device implantation), clinical complications (e.g., hypertension, diabetes mellitus), and medication intake (e.g., diuretics, beta-blockers) were collected before discharge from medical history.

Both PA and RHR were continuously measured and automatically transmitted every day by the remote monitoring system of CIED (Biotronik, Berlin, Germany). PA was measured using the acceleration sensors of CIED, and any acceleration above 0.473 m/s^2^ was recognized as activity. The value of daily PA was shown as the percentage in the remote monitoring system. For example, 1% means 0.24 h of daily PA and 10% means 2.4 h. Previous studies have proved that the acceleration sensors were highly sensitive in detecting PA (22–24). In order to better reflect the automatic nervous function, RHR was defined as the average value of heart rate from 2 o’ clock to 6 o’ clock at night, considering the influence of daily activities and daytime mood. Meanwhile, the target window for collecting PA and RHR was 30 to 60 days postoperatively in order to avoid the potential influence of operation on PA.

### Follow-up and outcome ascertainment

Daily PA and RHR were measured and automatically transmitted to the service center after CIED implantation. If data transmission is interrupted during the follow-up, clinical specialists will contact the patient and family members to confirm the monitoring function of the device and the condition of the patient. Also, routine telephone and outpatient follow-ups were conducted to collect clinical information about outcomes. The primary endpoint was all-cause mortality and the date of death was confirmed by medical records or death certificate.

### Statistical analysis

Categorical variables, which were presented as numbers with relative percentages, were compared using the chi-square test, whereas continuous variables, which were expressed as mean standard deviation, were compared between the groups using one-way analyses of variance. Box plots and Scatter plots were used to describe the distribution of RHR among different PA groups and assess the relationship between PA and RHR, respectively. Then, simple and multiple linear regression models were used to further confirm the trend. The independent predictive values of both PA and RHR for long-term all-cause mortality were assessed by univariable and multivariable Cox proportional hazards models. Variables with a *P* value of < 0.05 in the univariable models and other potential confounders were entered into the multivariable analysis [age at CIED implantation, gender, diabetes mellitus, stroke, ischemic cardiomyopathy (ICM), left ventricular ejection fraction (LVEF), left ventricular end-diastolic dimension (LVEDD), CRT-D implantation, diuretics usage, and aldosterone antagonist usage].

**FIGURE 1:**
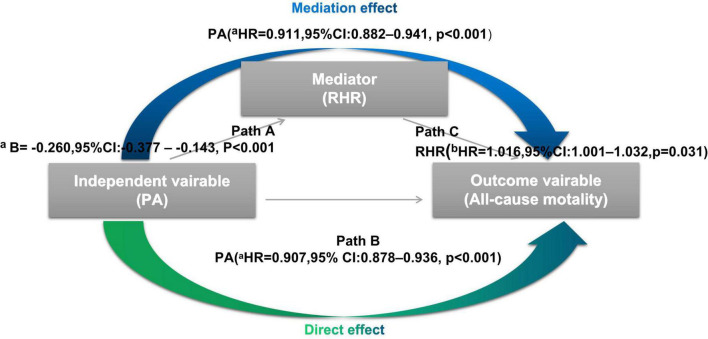
Processes of the causal mediation analysis In Path A, there is a significant linear relationship between physical activity (PA) (independent variable) and resting heart rate (RHR) (mediator) by the multiple regression model. In Path B, PA (independent variable) is shown as an independent predictor for all-cause mortality (outcome variables) in the multivariable Cox regression model without RHR. In Path C, RHR (mediator) can also be an independent predictor for all-cause mortality in the multivariable Cox regression model 2 where the mediator (e.g., PA) were included. CI, confidence interval; HR, hazard ratio; PA, physical activity; RHR, resting heart rate. ^a^Each additional 1% increase in PA. ^b^Each additional 1 beats increase in RHR.

According to Baron and Kenny’s (25) procedure, the causal mediation model was built to assess the possible mediation effect of RHR on the association between PA and all-cause mortality. As shown in Figure 1, there are three variables in the causal chains, including the independent variable (e.g., PA), mediator (e.g., RHR), and outcome variable (e.g., all-cause mortality). Causal mediation analysis allowed us to further explore the role of RHR in the impact of PA on all-cause mortality if the following three principles are met. Firstly, there is a linear relationship between the independent variable and the mediator in the multiple regression model (i.e., in Path A, changes of the independent variable account for the changes of the possible mediator). Secondly, the independent variable (e.g., PA) can predict the risk of the outcome variables (e.g., all-cause mortality) in the multivariable Cox regression model 1 without the mediator (e.g., RHR) (i.e., in Path B, changes of the outcome variables depend on the changes of the independent variable). Thirdly, the possible mediator (e.g., RHR) can also be an independent predictor for outcome variables (e.g., all-cause mortality) in the multivariable Cox regression model 2 where confounders are included in model 1 and additional mediators (e.g., PA) were included (i.e., in Path C, changes of the possible mediator significantly can be responsible for the outcome variables). In addition, the average causal mediation effect (ACME) and mediation proportion were further quantified by the R mediation package.

All statistical analyses were performed using IBM SPSS Statistics for Windows, version 23 (IBM Corp., Armonk, NY, USA) and R version 4.0.3 (Bunny-Wunnies Freak Out, The R Foundation for Statistical Computing, Vienna, Austria). Statistical significance was set at a *P* value < 0.05, and all tests were two-sided.

## Results

### Baseline characteristics and clinical outcome

**FIGURE 2:**
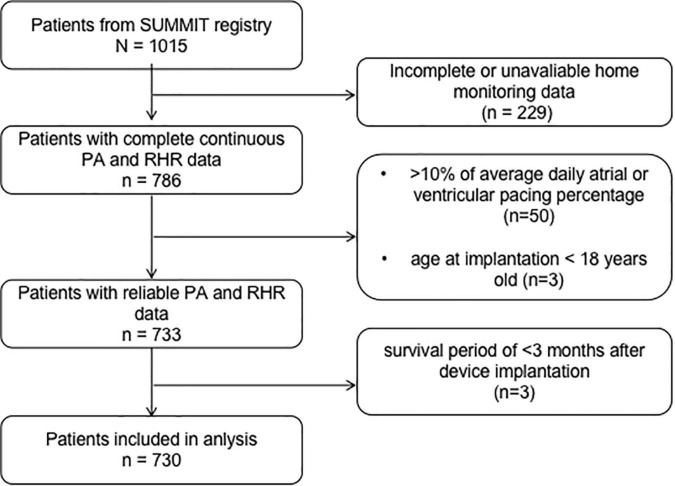
Flow chart for patient selection. PA, physical activity; RHR, resting heart rate.

Of the 1,015 patients who underwent CIED implantation with home-monitor function, 285 patients were excluded because of the following reasons: home monitoring data including PA or RHR were unavailable (*n* = 229); the average atrial pacing percentage > 10% or average ventricular pacing percentage in a dual-chamber device > 10% (*n* = 50), age at CIED implantation is younger than 18 years old (*n* = 3), and the postoperative survival is less than 3°months (*n* = 3). The flow chart is shown in Figure 2.

A total of 730 patients were included in the analyses and Baseline characteristics are shown in Table 1. The mean PA and RHR were 10.7 ± 5.7% and 61.3 ± 9.1 bpm, respectively. Based on the level of daily PA, patients were evenly divided into four groups: PA group 1 (range, 0.3–6.8%; *n* = 182), PA group 2 (range, 6.9–10.1%; *n* = 182), PA group 3 (range, 10.2–14.5%; *n* = 183), and PA group 4 (range, 14.5–33.3%; *n* = 183). The mean age of the participants at CIED implantation was 60.4 ± 13.9 years, 74.8% were men, 26.4% of patients received CRT-D implantation, 31.2% had hypertension, and 33.8% were ICM. The means of body mass index (BMI) and LVEF were 23.6 ± 3.0 kg/m^2^, and 42.8 ± 14.9%, respectively. Significant differences in the RHR (*p* < 0.001), age at CIED implantation (*p* < 0.001), LVEF (*p* = 0.03), history of diabetes (*p* = 0.002), stroke (*p* < 0.001), ICM (*p* < 0.001), diuretics usage (*p* = 0.008), and aldosterone antagonist usage (*p* = 0.005) were observed among different groups. During a mean follow-up period of 55.8 ± 22.7 months, 187 (26.5%) death was observed in the total cohort. With the daily PA increased, the rate of all-cause mortality in each group was gradually decreased (44.0% vs. 30.2% vs. 16.4% vs. 12.0%, *p* < 0.001).

**TABLE 1:** Baseline characteristics.

Parameters	Total (*N* = 730)	Group 1 (*n* = 182)	Group 2 (*n* = 182)	Group 3 (*n* = 183)	Group 4 (*n* = 183)	*P*-value
Home monitoring data						
PA, %	10.9 ± 5.7	4.2 ± 1.7	8.5 ± 1.0	12.3 ± 1.2	18.4 ± 3.9	-
RHR, bpm	61.3 ± 9.1	64.6 ± 10.0	61.4 ± 9.0	59.9 ± 8.0	59.3 ± 8.4	< 0.001
Demographics						
Gender, male	546 (74.8)	120 (65.9)	131 (72.0)	145 (79.2)	150 (82.0)	0.002
Age*, years	60.4 ± 13.9	66.3 ± 13.2	61.2 ± 14.1	58.9 ± 13.5	55.2 ± 12.6	< 0.001
BMI, Kg/m^2^	23.6 ± 3.0	23.4 ± 3.0	23.6 ± 3.4	23.5 ± 2.9	23.7 ± 2.8	0.817
CRT-D, %	193 (26.4)	52 (26.9)	47 (24.3)	46 (23.8)	48 (24.9)	0.904
Echocardiography						
LVEF, %	42.8 ± 14.9	40.3 ± 14.8	42.3 ± 14.8	44.5 ± 15.1	44.1 ± 14.8	0.03
LVEDD, mm	58.7 ± 13.2	58.8 ± 12.0	59.1 ± 13.5	58.6 ± 13.5	58.4 ± 13.8	0.97
Comorbidities						
Hypertension	228 (31.2)	63 (27.6)	61 (26.8)	58 (25.4)	46 (20.2)	0.204
Diabetes mellitus	76 (10.4)	31 (40.8)	21 (27.6)	15 (19.7)	10 (13.2)	0.002
Stroke	16 (2.2)	11 (68.8)	2 (12.5)	2 (12.5)	1 (6.2)	< 0.001
ICM	247 (33.8)	79 (35.3)	65 (26.3)	61 (24.7)	42 (16.0)	< 0.001
Paroxysmal AF	82 (11.2)	26 (31.7)	21 (25.6)	19 (23.2)	17 (20.7)	0.475
Medication						
ACEIs/ARBs	257 (35.2)	71 (27.6)	61 (23.7)	69 (26.8)	56 (21.8)	0.310
Diuretics	189 (25.9)	63 (33.3)	48 (25.4)	42 (22.2)	36 (19.0)	0.008
Aldosterone antagonists	260 (35.6)	83 (31.9)	63 (24.2)	63 (24.2)	51 (19.6)	0.005
Beta-blockers	413 (56.6)	100 (54.9)	94 (51.6)	113 (62.1)	106 (57.9)	0.248
Amiodarone	212 (29.0)	51 (24.1)	56 (26.4)	49 (23.1)	56 (26.4)	0.795

ACEIs, angiotensin-converting enzyme inhibitors; AF, atrial fibrillation; ARBs, angiotensin receptor blockers; BMI, body mass index; CRT-D, cardiac resynchronization therapy with defibrillation; ICM, ischemic cardiomyopathy; LVEF, left ventricular ejection fraction; LVEDD, left ventricular end-diastolic dimension; PA, physical activity; RHR, resting heart rate. *Age at the device implantation.

### The relationship between physical activity and resting heart rate measured by cardiac implantable electronic devices

**FIGURE 3:**
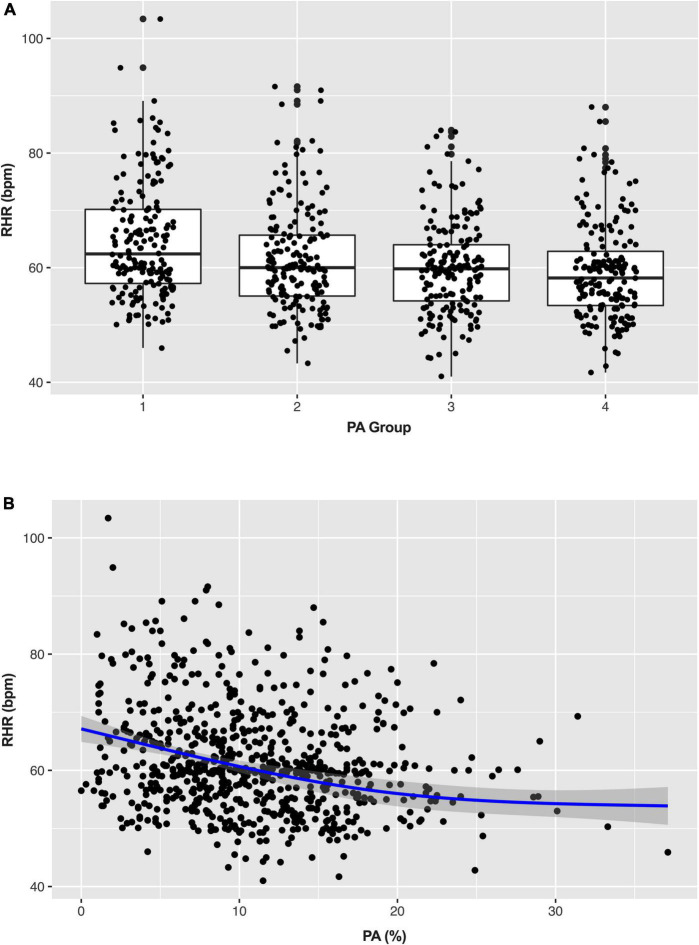
Box **(A)** and scatter plots **(B)** show the distribution of physical activity (PA) and resting heart rate (RHR). PA, physical activity; RHR, rest heart rate.

Figure 3A shows the distribution of RHR among different daily PA groups. The daily PA gradually increased from group 1 to 4 (4.2 ± 1.7% vs. 8.5 ± 1.0% vs. 12.3 ± 1.2% vs. 18.4 ± 3.9%), and the corresponding RHR decreased continuously (64.6 ± 10.0 bpm vs. 61.4 ± 9.0 bpm vs. 59.9 ± 8.0 bpm vs. 59.3 ± 8.4 bpm). As shown in the scatter plot (Figure 3B), there is an inverse linear relationship between PA and RHR. A simple linear regression model was further established to assess, and the β of RHR was −0.338 (95% CI: −0.452 to −0.225, *p* < 0.001). After adjustment for potential confounders (age, gender, LVEF, stroke, ICM, and β-blocker) in the multiple linear regression analysis, the β of RHR was −0.260 (95% CI: −0.377 to −0.143, *p* < 0.001) which means that RHR was decreased by 0.26 bpm for each additional 1% increase of daily PA.

### Predictive values of physical activity and resting heart rate for all-cause mortality

**FIGURE 4:**
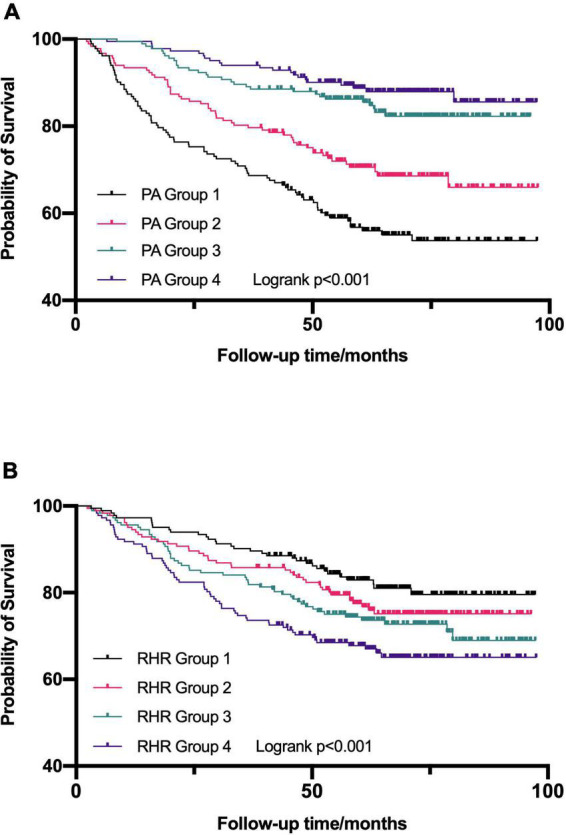
Kaplan–Meier survival curves were established to assess the predictive ability for all-cause mortality in the different quartile of daily physical activity (PA) **(A)** and resting heart rate (RHR) **(B)**. PA, physical activity; RHR, rest heart rate.

Estimated survival by the Kaplan-Meier method for the entire cohort stratified by quartile of daily PA (Figure 4A) and RHR (Figure 4B), respectively. In univariable Cox regression analyses, both daily PA and RHR by quartile were significantly associated with survival (Table 2). In univariable Cox regression model 1, a higher level of PA was related to reduced risks of all-cause mortality (HR = 0.907; 95% CI: 0.878–0.936, *p* < 0.001) after adjustment of possible variables (age at CIED implantation, gender, diabetes mellitus, stroke, ICM, LVEF, LVEDD, CRT-D implantation, diuretics usage, and aldosterone antagonist usage). In multivariable Cox regression model 2, adjusting for all confounders included in model 1 and additional RHR, daily PA was still a protective factor of all-cause mortality (HR = 0.911; 95% CI: 0.882–0.941, *p* < 0.001), illustrates that each additional 1% increase of daily PA can reduce the risk of all-cause mortality by 8.9%.

**TABLE 2:** Predictive values of physical activity (PA) and resting heart rate (RHR) for all-cause mortality outcomes.

All-cause mortality	Univariate		Multivariate (model 1)		Multivariate (model 2)	
			
	HR 95% CI	*P*-value	HR 95% CI	*P*-value	HR 95% CI	*P*-value
PA (1% increase)^a^	0.889 (0.862–0.916)	< 0.001	0.907 (0.878–0.936)	< 0.001	0.911 (0.882–0.941)	< 0.001
PA quantile 1 (ref.)	P-trend < 0.001		P-trend < 0.001		P-trend < 0.001	
PA quantile 2	0.614 (0.436–0.866)	0.005	0.716 (0.505–1.014)	0.060	0.753 (0.529–1.070)	0.114
PA quantile 3	0.305 (0.202–0.463)	< 0.001	0.376 (0.246–0.575)	< 0.001	0.400 (0.260–0.614)	< 0.001
PA quantile 4	0.202 (0.125–0.327)	< 0.001	0.271 (0.164–0.448)	< 0.001	0.288 (0.174–0.477)	< 0.001
RHR (1 bpm increase)^b^	1.037 (1.022–1.052)	< 0.001	1.024 (1.008–1.041)	0.003	1.016 (1.001–1.032)	0.031
RHR quantile 1 (ref.)	P-trend = 0.002		P-trend = 0.204		P-trend = 0.439	
RHR quantile 2	1.261 (0.797–1.994)	0.322	0.983 (0.617–1.567)	0.942	0.977 (0.614–1.553)	0.920
RHR quantile 3	1.621 (1.046–2.512)	0.031	1.206 (0.771–1.886)	0.412	1.180 (0.756–1.844)	0.466
RHR quantile 4	2.129 (1.396–3.249)	< 0.001	1.451 (0.932–2.258)	0.099	1.315 (0.849–2.037)	0.120

Multivariable Cox regression model 1 was adjusted for age at implantation, sex, LVEF, LVEDD, ICD, or CRT-D implantation, LVEF, DM, stroke, ICM, use of diuretics, and use of aldosterone antagonists. Multivariate Cox regression model 2 was adjusted for the above-mentioned confounders, as well as PA or HRV. CI, confidence interval; CRT-D, cardiac resynchronization therapy defibrillator; DM, diabetic mellitus; HR, hazard ratio; ICD, implantable cardioverter defibrillator; ICM, ischemic cardiomyopathy; LVEF, left ventricular ejection fraction; LVEDD, left ventricular end-diastolic dimension; PA, physical activity; RHR, resting heart rate. ^a^Each additional 1% increase in PA; ^b^Each additional 1 bpm increase in RHR.

Also, the univariable multivariable Cox regression analyses were established to further assess the predictive value of RHR on the risk of all-cause mortality (Table 2). Elevated RHR was related to higher risks of all-cause mortality (HR = 1.024; 95% CI: 1.008–1.041, *P* = 0.003) after adjustment for the confounders in model 1 (age at CIED implantation, gender, diabetes mellitus, stroke, ICM, LVEF, LVEDD, CRT-D implantation, diuretics usage, and aldosterone antagonist usage). After adjustment for the confounders mentioned in model 1 and additional PA (multivariable model 2), RHR as a continuous variable remained an independent predictor of all-cause mortality (HR = 1.016; 95% CI: 1.001–1.032, *P* = 0.031), indicating that each 1 bpm increase in RHR can account for a 1.6% increase in the risks of all-cause mortality. However, the association was weak in different RHR quantile groups.

### Mediation analysis

**FIGURE 5:**
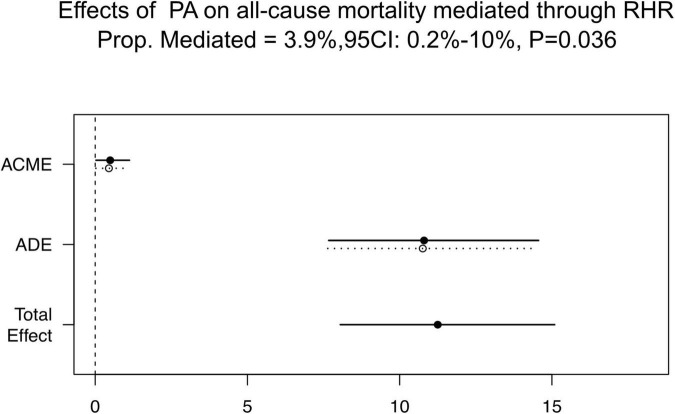
Causal mediation analysis results. ACME, average causal mediation effect; ADE, average direct effect; CI, confidence interval; PA, physical activity; RHR, rest heart rate; Prop. Mediated, mediation proportion.

As mentioned above, all three principles for causal mediation analysis have been met (Figure 1). In Path A, there is a significant linear relationship between PA (independent variable) and RHR (mediator) in the multiple linear regression model. Path B illustrated that PA (independent variable) was an independent predictor for all-cause mortality (outcome variables) using the multivariable Cox regression analysis without RHR involved. In Path C, RHR (mediator) remained significant in predicting all-cause mortality when PA (independent variable) was also included. As Figure 5 shown, mediation effect of RHR was statistically significant in the association of improved PA on reduced all-cause mortality (ACME = 0.49; 95% CI: 0.025–1.13, *P* = 0.036; total effect = 11.25; 95% CI: 8.05–15.09, *P* < 0.001), and the mediation proportion was 3.9% (prop. mediated = 3.9%; 95% CI: 0.2–10.0%, *P* = 0.036), indicating 3.9% effect of PA on the risks of all-cause mortality was mediated through RHR.

## Discussion

This retrospective analysis aimed to explore the relationship between RHR and PA which are continuously measured by CIED, and their value in predicting long-term all-cause mortality. The causal mediation analysis was established to verify the possible mediation effect of RHR on how PA improves all-cause mortality. The main findings were: (1) RHR was negatively related to PA, and both PA and RHR were independent predictors for long-term all-cause mortality; and (2) the mediation function of RHR was firstly confirmed and quantified in the process of PA improving all-cause mortality using causal mediation analysis for the first time, which may be critical to elucidate the potential mechanisms of the association between PA and all-cause mortality.

Clear evidence for the health benefits of PA began to emerge in the 1950s (5). Thereafter, the importance of regular PA to reduce mortality risks was gradually established (2–4). Although the possible reasons for the “PA paradox” were well-discussed recently (6–8), the heterogeneity among studies and the limitation of PA evaluation should not be ignored. PA was mostly assessed by using self-reported structured questionnaires in earlier research (6–12). The structure of each questionnaire with the evaluation latitude varies in different studies, which might contribute to the deviation of PA measurement. Recently, CIED was reported that could automatically collect information about PA through data analysis from equipped sensor accelerometers (22–24). Although the PA intensity measured by this method was limited by the preset threshold, it provided a quantitative, objective, easily accessible measure that may reflect individual functional status. The inverse relationship between device-measured PA and mortality was also identified among patients with heart failure (22). In accordance with previous studies, this study showed that every additional 1% increase in PA could result in an 8.9% reduction in risks of all-cause mortality.

Also, our findings support that elevated RHR is independently associated with increased all-cause mortality, since every additional 1 bpm increase in RHR could lead to a 1.6% increase in the risks of all-cause mortality. Previous research has focused on the relationship between RHR and prognosis, RHR was measured by palpating the radial pulse or during blood pressure monitoring and assessed at a single point after several minutes of rest (11, 12, 19, 20, 24). This may not accurately reflect the autonomic nervous function and physiological state, which may partly account for differences in the cut-off value of RHR among published studies (19, 20, 24). In this study, RHR was automatically and continuously collected by CIED sensors from 2:00 am to 6:00 am when patients are already asleep to avoid the influence of daily activities, work, and emotions on autonomic nervous function. Although the prognostic effect of RHR was not significant in subgroups, it did not affect the main result.

Given the methodological limitations of self-reported PA, as an indicator of physical fitness and general health, RHR might be an alternative measure of the level of daily PA (11, 12, 15). Emaus et al. (11) reported the association of RHR with PA measured by different methods. RHR was negatively associated with the increasing level of self-reported PA. This pattern was demonstrated in this study as well. As daily PA gradually increased, the corresponding RHR decreased continuously. After adjusting for potential confounding factors, significant negative and linear correlations between PA and RHR existed among patients with CIED. RHR was decreased by 0.26 bpm for each additional 1% increase in daily PA.

As discussed above, both PA and RHR have independent predictive values for all-cause mortality among patients with CIED, and they also have a linear relationship. Thus, causal mediation analysis was performed following Baron and Kenny’s (25) procedure. It showed that 3.9% (95% CI: 0.2–10.0%, *P* = 0.036) of the mediation effect of PA on the risks of all-cause mortality was mediated through RHR. This is the first study that verified and quantified the mediating effect of RHR on the progress of high PA improving long-term prognosis. The mechanism by which a high level of PA improves prognosis may partly be because of the low RHR induced by high PA. Specifically, the improvement in the autonomic nervous functions may be one of the reasons why regular PA decreased all-cause mortality. Therefore, RHR may be essential in providing insights into understanding the benefits of high levels of PA. Furthermore, changes in the autonomic nervous function during postoperative rehabilitation exercise should be considered among patients with CIED. Finally, when direct measures of PA levels are lacking, RHR may be used as an alternative to assess the relationship between PA and prognosis.

### Limitations

This study has some potential limitations that should be recognized. First, The mediation effect in this analysis is significant but relative low. It may indicate that the mechanism by which enhanced exercise improves prognosis is very complex, and the improvement of autonomic nervous function is only part of it. Data like PA and RHR were retrospectively collected and analyzed in this study. Prospective studies are needed to further quantify the mediation function of RHR. Second, the relationship between RHR and outcomes was weak in different RHR quartile groups. Although it does not affect the main results, a large sample and more detailed grouping methods were also needed to further explore differences in RHR subgroups. Third, although the accelerometer was highly sensitive in detecting whether a person is active or not, the intensity of PA measured was limited by the preset threshold. More accurate algorithms are needed to further distinguish the levels of PA. In addition, all selected patients were those with ICD or CRT-D and underwent a high risk of sudden cardiac death, which may cause potential effects on daily PA and RHR. Thus, generalizing the findings to other populations requires more caution.

## Conclusion

The findings of this study show that both PA and RHR, continuously measured by CIEDs, were independent predictors for long-term all-cause mortality. Moreover, causal mediation analysis further verified that the partial mediation effects of PA on improving life prognosis were mediated through RHR. It indicates that the changes in autonomic nervous function may be an important step in the process of improving the prognosis by regular PA. Changes in the autonomic nervous function during postoperative rehabilitation exercise should get more attention among patients with ICD/CRT-D.

## Data availability statement

The raw data supporting the conclusions of this article will be made available by the authors, without undue reservation.

## Ethics statement

The studies involving human participants were reviewed and approved by National Center for Cardiovascular Diseases, Fuwai Hospital, Chinese Academy of Medical Sciences, Peking Union Medical College, Beijing, China. The patients/participants provided their written informed consent to participate in this study.

## Author contributions

CC and XS had the idea for the study. CC wrote the draft report. SZ and XS revised the manuscript. All authors contributed to the study design, data interpretation, read, and approved the final manuscript.
